# Understanding the Experiences of end of Life Care for Patients with Mesothelioma from the Perspective of Bereaved Family Caregivers in the UK: A Qualitative Analysis

**DOI:** 10.1177/08258597221079235

**Published:** 2022-02-16

**Authors:** Madeleine Harrison, Liz Darlison, Clare Gardiner

**Affiliations:** 1 The University of Sheffield, Sheffield, UK; 2 University Hospital of Leicester NHS Trust, The Glenfield Hospital, Leicester, UK; 3 Mesothelioma UK, Leicester, UK

**Keywords:** mesothelioma, family caregivers, palliative care, end-of-life care, qualitative research, informal caregivers

## Abstract

**Objectives:** Mesothelioma is a rare, progressive cancer with a short trajectory from diagnosis to the end of life. This study explores the experiences of palliative and end of life care for patients with mesothelioma from the perspective of bereaved family caregivers. **Methods:** A qualitative, descriptive approach was adopted comprising face-to-face, semi-structured interviews with bereaved caregivers of patients with mesothelioma in the UK. An inductive, thematic analysis was conducted using the ‘Framework’ approach. **Results:** Nine bereaved caregivers participated. Four themes emerged: understanding what lies ahead; carer support; care co-ordination; managing after death: practicalities, inquests and abandonment. Caregivers need to understand what lies ahead in order to emotionally and practically prepare themselves for supporting the patient at the end of life. Information and support needs of caregivers were often distinct from those of patients, including the importance of information about the coroner's involvement. The importance of care co-ordination was emphasised, with caregivers valuing on-going relationships and a named individual taking responsibility for co-ordinating the patients care. Feelings of abandonment arose when there was no contact with healthcare professionals following the death of the patient. **Conclusions:** Greater opportunity for open, one-to-one communication between family caregivers and healthcare professionals is vital to enable the caregiver to prepare for what lies ahead at the end of life and prevent feelings of abandonment after the death of the patient. Improved care co-ordination and partnership working are essential for supporting both patient and caregiver at the end of life.

## Introduction

Mesothelioma is an aggressive malignancy of the pleura or peritoneum. The disease has a high symptom burden, including: breathlessness, cough, pain, fatigue, weight loss, anxiety and low mood.^1,2^ There are approximately 2700 new cases of mesothelioma diagnosed in the United Kingdom (UK) each year.^3^ Most cases are caused by preventable asbestos exposure, which usually occurs in the workplace.^4^ Due to the long latency period, the condition typically occurs in older people and predominantly in men.^4–6^ No curative treatments are currently available for mesothelioma, but chemotherapy, radiotherapy, surgery, and more recently immunotherapy, can extend survival and palliate symptoms.^7^

Mesothelioma has a relatively short disease trajectory, with patients surviving on average one year from diagnosis.^3^ Consequently patients and their families have palliative care needs from the time of diagnosis with mesothelioma through to the end of life and bereavement.^8^ The challenge of delivering palliative care to these patients is growing as the global number of mesothelioma deaths are increasing; recently estimated to be 38 400 per year globally.^9^ The British Thoracic Society and the European Respiratory Society emphasise the value of high quality palliative care to manage the symptoms of mesothelioma and provide emotional, psychological and spiritual support.^10,11^ However, patient and carer surveys indicate that end of life provision is variable with less than one third of mesothelioma patients referred to specialist palliative care in Australia^12^ and only 40% of patients who wanted support to plan for end of life care actually receiving it in the UK.^13^

A recent multicentre randomised controlled trial evaluated routine early referral to specialist palliative care for patients with mesothelioma.^14^ Following trials in similar patient groups that have demonstrated quality of life benefits of such interventions,^15^ the mesothelioma trial demonstrated no quality of life benefits of early specialist palliative care to patients.^14^ However, carer satisfaction with end of life care was significantly higher in the early referral group with notable improvements in the emotional support provided to the patient and their family members, attention to the patient's symptoms and response to changes in symptoms.

Family and friends have been identified as a key source of support for people diagnosed with mesothelioma.^12^ Providing support and care for the patient requires the family caregiver to assume a significant emotional and physical burden, particularly toward the end of life.^16^ There is limited existing research evidence exploring the experience of end of life care for patients with mesothelioma, and even less information from the perspective of family caregivers.^12–14,17,18^ However, evidence from carers of patients with other cancers highlights issues including a lack of practical support, inadequate information exchange particularly around practically-focussed tasks, and significant impacts on carer wellbeing and confidence.^19^ End of life caregivers have reported that, to maintain their health and well-being, they need adequate resources, fairer remuneration, quality respite care, education about the role, equipment, psychosocial support, and improved access to the paid workforce.^20^ There is no existing research focussed specifically on the experience of bereaved caregivers of patients with mesothelioma. Evidence from caregivers surveyed earlier in the mesothelioma trajectory indicates that many would like clearer information about mesothelioma, the opportunity to talk to someone by themselves,^12,18^ more psychological support, and help with planning for the end of life.^13^ The role of palliative and end of life care in mesothelioma is central, yet questions remain about when and how this should be introduced, and what the palliative care needs of caregivers are. Consequently, the aim of this study was to fill that gap by exploring the experiences of palliative and end of life care for patients with mesothelioma from the perspective of bereaved family caregivers.

## Methods

### Study Design

We adopted a qualitative descriptive approach^21^ with semi-structured interviews conducted with bereaved caregivers of patients with mesothelioma. A qualitative approach was chosen to capture the multi-dimensional experience associated with mesothelioma end of life care, and to provide a deep understanding of this experience as uniquely constructed by those involved. Ethical approval was obtained from the Health Research Authority and East Midlands research ethics committee (REC 13/EM/0038). The COREQ checklist (COnsolidated criteria for REporting Qualitative research) was used as the reporting guideline.^22^

### Participants

Participants were recruited from a large acute (hospital) NHS Trust in the East Midlands of England, during 2014.Specialist palliative care services are provided across the region by in-hospital teams, community palliative care teams and hospice. This broadly reflects the provision of specialist palliative care across the UK. Participants were eligible for inclusion if they were primary caregivers of patients with mesothelioma who had been bereaved between 6 and 24 months prior to recruitment. Caregivers were identified from past patient records and invited to participate by letter from their mesothelioma nurse. We recruited bereaved caregivers rather than current caregivers as they are able to reflect on the whole mesothelioma journey from diagnosis to end of life and bereavement. Sample size was dictated partly by pragmatism (recruiting within a given timescale) and partly by considerations of saturation. In this context we refer to saturation as sufficient interviews to get a reliable sense of thematic exhaustion and variability within our data set. Using this definition recruitment stopped once data saturation was achieved.^23,24^

Seventeen caregivers were contacted, and of these nine agreed to participate. All participants had been the main carer for their spouse or partner who died with mesothelioma between 12 and 22 months prior to the interview (table 1).

**Table 1.** Demographic Characteristics of Bereaved Carers and Patients.

Gender of carer	Male	n = 1
	Female	n = 8
Gender of patient	Male	n = 9
Patient survival from diagnosis	Range: 4–160 weeks	
Patient place of death	Hospital	n = 3
	Home	n = 5
	Hospice	n = 1
Patient expressed preferred place of death	n = 6	
Patient died in preferred place of death	n = 5	

### Data Collection

Caregivers were interviewed face-to-face by LD (a mesothelioma nurse) as part of a Masters project. The interview guide comprised open-ended questions to explore caregivers’ experiences and perspectives regarding the patients end of life care and support (table 2). The development of the questions was informed by members of the National Lung Cancer Forum for Nurses - Research Interest Groups and piloted with a bereaved caregiver. All participants provided written informed consent prior to participation. Interviews were conducted in a place convenient for the caregiver (home or outpatient clinic) and ranged in length from 40 to 92 minutes. The interviews were digitally audio recorded and transcribed verbatim. Participants were offered a copy of the transcription and an informal follow-up phone call one week after the interview.

**Table 2.** Interview Topic Guide.

**Interview topic guide**
Can you tell me a little bit about how things were when [patient name] was really poorly, in the days and weeks leading up to his/her death? Did you have any conversations with anyone leading up to [patient name] last few days/weeks? Did you feel prepared to look after [patient name]? (emotionally/practically) Was support from carers, doctors and nurses forthcoming? What support was missing? What was most frustrating? In terms of [patients name] care what worked particularly well? If you had to do it again what would you have changed? What advice would you give to someone else facing the same situation? What if anything would have made the situation easier to experience? Before we switch the tape off is there anything you would like to add?

### Analysis

An inductive analysis was conducted using the ‘Framework’ method. This method sits within a broad family of analysis methods often termed thematic analysis which identify commonalities and differences in qualitative data, seeking to draw descriptive conclusions clustered around themes.^25^ This analysis involved reviewing the data (reading and re-reading) and coding the transcripts in order to identify recurrent and important themes. Analysis begun after the first interview in order to guide decisions on saturation and sample size. This resulted in a thematic index, developed by LD, which was used to label the interview data. The full range and diversity of coded data was then summarised and synthesised by refining initial themes and categories, and was displayed in a thematic matrix in Excel. This method of presentation enabled exploration of patterns within the data and associations between the themes, allowing the development of more abstract concepts.^26^ MH independently read all transcripts and contributed to the process of interpretation for this paper.

## Results

Demographic characteristics of the participants are provided in table 1. Four themes emerged from the data: understanding what lies ahead; carer support; care co-ordination; managing after death: practicalities, inquests and abandonment.

### Understanding What Lies Ahead

In order to prepare themselves to care for their loved one at the end of life, caregivers expressed a need to understand what lies ahead. Effective communication between health care professionals and the patient and/or carer was crucial to understanding that the end of life was approaching. However, effective communication did not always refer to death directly, for example, healthcare professionals would suggest addressing practical issues or stopping treatment in order to convey that the patient was nearing the end of life. Caregivers described wanting more information than patients around length of expected survival and what would happen at the end of life. They also felt there was little opportunity to communicate with healthcare professionals without the presence of the patient, and when the opportunity arose it was often under less than ideal circumstances.

The doctor asked me had he made a will and was his finances in order so I knew then. C1

We were whispering and he was just behind the screen sort of thing, I said how many months do you think…oh I don’t know six…I don’t know if he heard. I was stunned. C2

Caregivers also felt that understanding the patient's individual priorities and preferences was important, without this carers often felt unprepared. When they occurred, open and honest conversations between patients and carers about their hopes and expectations for the end of life were valued.

I was not prepared for how he coped with it, he didn’t fight it at all, and it was out of character right from the start. It will always be my biggest disappointment really. C2

I suppose we are both really open and he was not scared to talk about it and one day he said to me ‘do you think I will be here at Easter?’ and I said well you are not really eating anything so I don’t think so. I mean people wouldn’t normally say that although to me honesty and being able to talk about it is important. C8

### Carer Support Needs

Caregivers of patients with mesothelioma recognised that their support needs were distinct to those of the patient. Friends and neighbors were a key source of emotional and practical support to all caregivers involved in the study. While family support was vital to some, others highlighted the need for external (non-familial) support because they did not want to burden family, especially their children. Support groups were considered vital to those who joined them, these included Asbestos Support Groups (ASG's) which provide not only support but also advice on benefits and compensation. Howeverit was acknowledged that they were not suited to everyone and some participants had support needs met informally.

I had a good friend at the time, I think if she hadn’t been there in those first few months, well, we talked, she helped a lot. I could talk to her about anything over those few months and I felt a lot better just talking to her. C4

Carers described mixed experiences of the support they received during patients’ hospital admissions. In cases where patients were admitted to non-cancer wards there was a perception that staff lacked understanding of patients’ palliative care needs or carers’ desire to remain with the patient in their final days.

He ended up in a…ward and he felt he was totally in the wrong place in the sense that he was the only cancer patient in the ward. They kept insisting he have physiotherapy, but I kept telling them he was too weak. C5

### Care Co-Ordination and Continuity

Caregivers’ often described frustrations with disjointed care and highlighted the importance of care co-ordination between the various providers involved in palliative care. Where a single person was responsible for co-ordinating the patients care this was highly valued, particularly from community nursing teams. However, caregivers more frequently described fragmented care. The complexity of the care teams and communication channels often led to confusion regarding who the caregiver should contact, particularly when accessing services out of hours, when the quality of care was perceived as more variable. These issues are exacerbated in mesothelioma which is a rare cancer, where specialist expertise is less common and some health professionals may have little knowledge or experience of the condition.

We had a wonderful Macmillan [specialist palliative care] nurse but she left and we were both devastated by that. We felt very let down, they didn’t replace her so we would get a different Macmillan nurse each time, you just didn’t see one often enough to form a bond, and they didn’t come out anywhere near as much. I am sure if [specialist palliative nurse] had stayed it wouldn’t have been as bad as it was. C5

The people called out in the middle of the night were not singing from the same song sheet as the district nurses. C7

Poor communication particularly regarding practicalities such as care equipment and supply of medicines resulted in distress and frustration. Carers often felt they had to take on the role of patient advocate and be quite forceful to ensure their loved one received appropriate and timely care.

I said you’re not touching him, I said you leave his dressing alone, I told them I didn’t want him messing about. I said that was the fifth time that we had problems and I do not want it happening. C9

### Managing After Death: Practicalities, Inquests and Abandonment

Caregivers spoke about their experiences after the death of their loved one. Particular difficulties were noted when the patient died in their own home, as most caregivers were not prepared for dealing with the practicalities of what to do.

I asked the GP, I said look I need to know what I need to do if anything happens. She told us what to do, about getting the out of hours GP in. I never gave an undertaker a second thought; I don’t know what I thought really I just hadn’t got that far. Having an information booklet ahead of time about all this would have been helpful. C9

As mesothelioma is an industrial disease, patients and their carers face some unique issue. These include potentially protracted legal claims for compensation, and the prospect of a coroners inquest after the patients death (in the UK, a coroner's inquest is required by law in any death from an industrial disease, including mesothelioma). The majority of participants were made aware of the coroner's involvement and commented that this information had helped them to prepare for what was to come, and those who were not informed wished they had been. For some carers, the civil (legal) claims process was highlighted as an ongoing cause of distress that continued after the death of their loved one.

I knew about the coroner and inquest and it was really helpful that I did. C2

It has been very difficult because I’ve had to go over and over and over and correct things and then go over and over again and we haven’t been able to close the door. C1

Some caregivers described a lack of insight into grief and feelings of abandonment after the patient died. Several caregivers felt they would have benefitted from information about the stages of grief. Feelings of abandonment arose when contact with health care professionals abruptly ceased after the patient's death. Some felt they would have benefitted from bereavement support or contact with a member of the heath care team after the death of the patient.

They were like friends for all those weeks on end and it's just as though everything's dead and you know it really upset us. We didn’t get a visit, a card or a phone call from any of them. That really upset us. C9

## Discussion

Our results describe the experiences of UK family caregivers of patients with mesothelioma towards the end of life and highlight a range of challenges and support needs. Four key themes were identified – understanding what lies ahead, carer support needs, care co-ordination and managing after death. The themes are individually distinct but conceptually can be considered as important components of good palliative care. Palliative care has a holistic remit to support patients and their families and improve quality of life across the illness trajectory.^19,20^ Our data on the experience of carers supports the important role of palliative care in mesothelioma from diagnosis to the end of life and bereavement. Understanding what lies ahead and caregiver preparedness for what to expect at the end of life were identified as key needs, a finding in line with evidence from other cancers. Studies on carer needs in advanced cancer and other palliative care populations have identified insufficient carer preparedness as impacting negatively on quality of life.^20,27^ Caregivers need more opportunities for effective communication with healthcare professionals to understand what to expect toward the end of the patients’ life and after their death. However, a UK survey from 2014 found that 71% of mesothelioma patients and carers did not receive support with end of life care planning.^13^ Data from our study supports these findings and highlights the importance of effective communication from healthcare professionals and between patients and caregivers around end of life issues. However, our findings indicate that effective communication does not necessarily mean direct discussion about end of life and death, which can be distressing for some mesothelioma patients.^28,29^ Sensitively constructed discussions, tailored to the individual needs of patients and carers, can be effective in supporting these patients. The importance of providing the opportunity for separate one-to-one discussions with the caregiver around end of life matters was also highlighted. Evidence suggests that interventions targeting the caregiver, for example a psychoeducation component delivered by nurses,^30^ can be effective at improving carer preparedness.

Palliative and end of life care were typically perceived to be fragmented, involving complex care teams and multiple specialities, resulting in repeated hospital admissions and variable access to community services. Caregiver confidence appeared to be improved when a specific individual was perceived to be responsible for co-ordinating the patients care, particularly where the individual was accessible and a relationship had developed over time. This supports the findings of a literature review which identified that enhanced access to professional advice has the potential to increase carers’ confidence in their ability to undertake practical aspects of home-based care at the end of life.^19^ Clinical nurse specialists may be well placed to support continuity of care in mesothelioma, given their specialist expertise and integration across primary and secondary care.^31^ A lack of co-ordinated care has profound implications for the caregiver who may be forced, sometimes reluctantly, to take on the role of patient advocate, as has been seen more widely in end of life care for patients with advanced cancer.^19,20^

Our findings highlight the need for better preparation and bereavement support for caregivers after a patient's death. Improved preparedness for death has been found to reduce bereavement-related distress.^32^ One element of preparedness and bereavement support unique to mesothelioma relates to the coroners inquest (and sometimes a post-mortem) which is required for deaths from industrial disease.^13^ The majority of participants involved in the study were aware of the coroners involvement and felt it was valuable to have received information about this in advance. However, bereavement support was variable and feelings of abandonment arose for caregivers when there was no opportunity for contact with healthcare professionals after the patient's death, particularly where a relationship has been built in the course of caring for the patient. This supports findings from previous research which indicate many bereaved caregivers of patients with mesothelioma want a post death consultation.^12^ Whose responsibility it is to provide such support may vary, but could involve clinical nurse specialists in collaboration with formal bereavement services from charities or other organisations.

### Limitations

The interviewer was known to the patients in a clinical capacity (but with no involvement in end of life care), which might have impacted upon their willingness to share negative experiences. To minimise this anonymity was guaranteed and participants were encouraged to share both their positive and negative experiences. It is likely that participant's accounts will have been impacted by recall bias as they were recruited between six and 22 months after their bereavement. Data were not collected on some participant demographics such as patient occupation, age and level of education which may have been useful in interpreting findings. The findings may have limited international transferability because all participants were recruited from one hospital in the East Midlands of England. The caregivers in the study were all caring for male patients, however this is reflective of the higher prevalence of mesothelioma in men.^33^

## Conclusion

This study affirms the importance of providing comprehensive support for family caregivers of patients with mesothelioma, findings which are likely to be transferable to other advanced cancers. Family carers should be given the opportunity for open, one-to-one communication with healthcare professionals in order to prepare for supporting the patient at the end of life. Improved care co-ordination is vital and caregivers value on-going relationships with accessible health care professionals. Clinical nurse specialists may be well placed to support family caregivers and improve continuity of care across the patient pathway and into bereavement. Further research may be required to explore the most effective strategies for supporting carers throughout the illness trajectory and beyond, potentially making use of tools developed for other populations (eg the Carer Support Needs Assessment Tool or CSNAT).^34^ Improved carer support is crucial and is associated with improved well-being and confidence in their ability to provide care at the end of life and manage after the patient's death.

## Supplementary Material

Supplementary material
